# Suggestive

**Published:** 1868-05

**Authors:** 


### SUGGESTIVE.

The time of the annual meeting of the American Dental Association is approaching and will soon be here, and we trust it will be one profitable to all who may take part in its proceedings, and be largely productive of good to the whole profession, as it may, and will be, if properly conducted, and the work earnestly engaged in.

Such associations should become more and more efficient for good. Experience should always secure improvement, if not perfection. Difficulties met and overcome should impart new vigor, strength and wisdom. Rocks once encountered should serve as warnings in a future course. We hope the American Dental Association will improve from the experience of its former meetings, avoiding those things that proved to be unwise and injurious, in their tendency, and using those things that are desirable and advantageous in their results. The particular points of professional matters upon which the time of the Association can be most profitably spent we will not now attempt to specify.

Bearing upon this point, we give place to the following remarks from "*The Boston Medical and Surgical Journal*," in reference to the American Medical Association, after its recent meeting. The suggestions therein contained apply in many particulars as well to the American Dental Association, as the Medical. We think, however, that by a judicious course, practical details may be more fully developed in the former than in the latter, and from the fact that manipulation enters more largely into its practice.

T.

### MEETING OF THE AMERICAN MEDICAL ASSOCIATION.

Our national medical society has just held its annual meeting. Socially a success, it can hardly be considered so as a scientific re-union. Nor can we ever reasonably expect it to be otherwise. Its influence in removing sectional prejudices, in familiarizing the physicians of one part of our extended country with other parts, and with their professional brethren, is both great and salutary. It is, however, too widely extended, and holds too infrequent and too brief meetings to render its scientific proceedings choice or valuable. The great majority of our physicians are too busy in solving the practical problems of life and death daily presented to them, or too much occupied in the pursuit of individual aggrandizement and reputation, to become *savants*, or to devote themselves to the cultivation of pure science. It is a pity, too, that those unqualified to speak instructively, and those who delight in parliamentary quibbles, are allowed to take up the valuable time of a three day's annual session. Such, however, is the fate of many other large societies. The International Medidal Congress in Paris was a signal instance of a like failure. The efforts of the National Association to raise the standard of Medical requirements cannot be too highly praised; and it is by this means only that the Society itself can ever become worthy of representing the whole nation.

