# Correction

**Published:** 1868-06

**Authors:** 


### CORRECTION.

In the notice of the Executive Committee of the American Dental Association, published in the May number of the Register, by an oversight the location of that Committee was omitted. All the members of the Committee reside at Buffalo, N. Y.

